# Reply to Dion et al. Comment on “Sun et al. First Experience with Hypothermic Oxygenated Perfusion in Human Uteri: Feasibility and Metabolic Characterization. *J. Clin. Med.* 2026, *15*, 2820”

**DOI:** 10.3390/jcm15124696

**Published:** 2026-06-17

**Authors:** Keyue Sun, Nasim Eshraghi, Fernanda Walsh Fernandes, Sangeeta Satish, Chunbao Jiao, Fatma Selin Yildirim, Geofia Crasta, Omer F. Karakaya, Koki Takase, Hiroshi Horie, Karen S. Keslar, Dylan Isaacson, William Baldwin, Robert L. Fairchild, Koji Hashimoto, Alejandro Pita, Alvin Wee, Mariam AlHilli, Charles Miller, Mohamed Eltemamy, Tommaso Falcone, Andreas Tzakis, Elliot Richards, Andrea Schlegel

**Affiliations:** 1 Department of Inflammation and Immunity, Cleveland Clinic Research Institute, Cleveland, OH 44195, USA; eshraghinasim44@gmail.com (N.E.); fernandawalshfernandes@gmail.com (F.W.F.); satishs2@ccf.org (S.S.); jiaoc@ccf.org (C.J.); yildirf@ccf.org (F.S.Y.); crastag2@ccf.org (G.C.); karakao@ccf.org (O.F.K.); takasek@ccf.org (K.T.); horieh@ccf.org (H.H.); keslark@ccf.org (K.S.K.); baldwiw@ccf.org (W.B.); fairchr@ccf.org (R.L.F.); 2 Transplantation Center, Digestive Disease and Surgery Institute, Cleveland Clinic, Cleveland, OH 44195, USA; hashimk@ccf.org (K.H.); pitaa@ccf.org (A.P.); millerc8@ccf.org (C.M.); tzakisa@ccf.org (A.T.); 3 Department of Urology, Glickman Urological and Kidney Institute, Cleveland Clinic, Cleveland, OH 44195, USA; isaacsd3@ccf.org (D.I.); weea@ccf.org (A.W.); eltemam@ccf.org (M.E.); 4 Department of Obstetrics, Gynecology and Reproductive Biology, Cleveland Clinic, Cleveland, OH 44195, USA; alhillm@ccf.org (M.A.); falcont@ccf.org (T.F.); 5 Wake Forest Baptist Center for Fertility and Reproductive Surgery, Atrium Health, Winston Salem, NC 27103, USA; elliott.richards@advocatehealth.org

We thank Dion et al. [1] for their careful reading of our manuscript and for raising the question of arterial perfusion configuration in uterine HOPE. Their group has made meaningful contributions to this field, and we welcome this exchange. Below, we address the technical considerations they raised given the available anatomical and experimental evidence.

## 1. Patency Was Confirmed Before Perfusion

Each organ received an individual 1 L cold HTK back-table flush prior to HOPE initiation, allowing direct visualization of inflow patency from each side and confirmation of bilateral vascular integrity before machine perfusion began [2]. Should individualized arterial assessment be required during perfusion, selective evaluation is readily achievable by temporarily clamping one limb of the Y-connector to isolate each side independently, without requiring a dual-pump configuration.

## 2. Uterine Cross-Perfusion Provides an Additional Layer of Resilience

The uterine arteries give rise to arcuate arteries that course circumferentially through the myometrium and form extensive midline anastomoses between right and left sides [3]. Kristek et al. (2022) demonstrated this directly by injecting India ink into a single uterine artery in human specimens: dye permeated beyond the midline in all cases (9/9, 100%), with near-complete or complete contralateral distribution in 66% [4]. While transmural myometrial penetration was complete in only 22% of specimens, contralateral surface distribution was consistent across all cases, supporting the existence of a cross-perfusion network that, together with our bilateral flush assessment and fluorescein confirmation, substantiates adequate perfusion for the objectives of our feasibility study.

## 3. Homogeneous Perfusion Was Confirmed Prospectively

As a quality assessment step, fluorescein imaging was performed in one uterus as a proof of concept. As reported in our original publication (Figure 1e), this confirmed rapid and homogeneous tissue perfusion by both macroscopic and microscopic examination, with fluorescein positivity across vascular structures and cells in all uterine tissue layers [2]. Perfusion was delivered via dual cannulation of the bilateral internal iliac arteries at 15 mmHg, with stable flows of 70–150 mL/min maintained for 8 h across all organs without technical issues [2].

We agree with Dion et al. that perfusion homogeneity and accurate organ assessment are paramount, and we wish to clarify that we do not argue against the importance of bilateral arterial inflow in clinical uterus transplantation, where vascular patency is directly linked to graft survival. Our findings pertain specifically to ex situ hypothermic oxygenated perfusion (HOPE) and metabolic characterization, a setting with fundamentally different objectives and perfusion requirements. As the first reported series of HOPE in human uteri, no adverse perfusion-related findings were observed across all organs, offering preliminary human evidence that this configuration was well tolerated in practice. In this context, the combination of individual back-table flush assessment, the option of selective Y-limb clamping for real-time arterial evaluation, fluorescein confirmation of homogeneous distribution, and the established contralateral anastomotic network of the uterus collectively address the concerns raised. We acknowledge that whether cross-perfusion provides functionally adequate flow under hypothermic low-pressure conditions remains an open question worth investigating. We also note that most dual-perfusion studies in uteri were applied under normothermic rather than hypothermic conditions [5], representing a distinct use case. Prospective comparative studies on this topic are welcomed.

## References

[1] Dion L., Sousa C., Dugast M., Lavoué V. (2026). Comment on Sun et al. First Experience with Hypothermic Oxygenated Perfusion in Human Uteri: Feasibility and Metabolic Characterization. *J. Clin. Med.* 2026, *15*, 2820. J. Clin. Med..

[2] Sun K., Eshraghi N., Fernandes F.W., Satish S., Jiao C., Yildirim F.S., Crasta G., Karakaya O.F., Takase K., Horie H. (2026). First Experience with Hypothermic Oxygenated Perfusion in Human Uteri: Feasibility and Metabolic Characterization. J. Clin. Med..

[3] Standring S., Ellis H., Healy J., Johnson D., Williams A., Collins P., Wigley C. (2005). Gray’s Anatomy, 39th Edition: The Anatomical Basis of Clinical Practice. Am. J. Neuroradiol..

[4] Kristek J., Kachlik D., Sticova E., Fronk J. (2022). Contralateral and Ipsilateral Arterial Vasculature of the Human Uterus: The Pilot Results of an Anatomical Study. Physiol. Res..

[5] Drivas E.M., Khaki S., Loftin A.H., Lamsehchi N., Johannesson L., Oh B.C., Brandacher G. (2025). A Comprehensive Review of Ex-Vivo Machine Perfusion in Uterus Transplantation. Transpl. Int..

